# Ubiquitin-proteasome system-based signature to predict the prognosis and drug sensitivity of hepatocellular carcinoma

**DOI:** 10.3389/fphar.2023.1172908

**Published:** 2023-04-25

**Authors:** Jianxiang Zhang, Liwen Liu, Zenghan Wang, Mingyang Hou, Zihui Dong, Jia Yu, Ranran Sun, Guangying Cui

**Affiliations:** ^1^ Department of General Surgery Department, The First Affiliated Hospital of Zhengzhou University, Zhengzhou, China; ^2^ Precision Medicine Center, The First Affiliated Hospital of Zhengzhou University, Zhengzhou, China; ^3^ Department of Pharmacy, The First Affiliated Hospital of Zhengzhou University, Zhengzhou, China

**Keywords:** hepatocellular carcinoma, ubiquitin-proteasome system, drug sensitivity, risk model, prognosis

## Abstract

**Background:** Ubiquitin-proteasome system (UPS) is implicated in cancer occurrence and progression. Targeting UPS is emerging as a promising therapeutic target for cancer treatment. Nevertheless, the clinical significance of UPS in hepatocellular carcinoma (HCC) has not been entirely elucidated.

**Methods:** Differentially expressed UPS genes (DEUPS) were screened from LIHC-TCGA datasets. The least absolute shrinkage and selection operator (LASSO) and stepwise multivariate regression analysis were conducted to establish a UPS-based prognostic risk model. The robustness of the risk model was further validated in HCCDB18, GSE14520, and GSE76427 cohorts. Subsequently, immune features, clinicopathologic characteristics, enrichment pathways, and anti-tumor drug sensitivity of the model were further evaluated. Moreover, a nomogram was established to improve the predictive ability of the risk model.

**Results:** Seven UPS-based signatures (ATG10, FBXL7, IPP, MEX3A, SOCS2, TRIM54, and PSMD9) were developed for the prognostic risk model. Individuals with HCC with high-risk scores presented a more dismal prognosis than those with low-risk scores. Moreover, larger tumor size, advanced TNM stage, and tumor grade were observed in the high-risk group. Additionally, cell cycle, ubiquitin-mediated proteolysis, and DNA repair pathways were intimately linked to the risk score. In addition, obvious immune cell infiltration and sensitive drug response were identified in low-risk patients. Furthermore, both nomogram and risk score showed a significant prognosis-predictive ability.

**Conclusion:** Overall, we established a novel UPS-based prognostic risk model in HCC. Our results will facilitate a deep understanding of the functional role of UPS-based signature in HCC and provide a reliable prediction of clinical outcomes and anti-tumor drug responses for patients with HCC.

## Introduction

Hepatocellular carcinoma (HCC) is the most frequent type of liver neoplasm, with a global high incidence and mortality rate (Sung et al., 2021). Notably, the incidence of HCC will rise greatly in the future due to excessive drinking, viral hepatitis infection, and emerging fatty liver diseases (Kirstein and Vogel, 2014). Although the early diagnosis of HCC has been improved, patients usually present pathognomonic symptoms (Mao et al., 2019). Nowadays, surgery intervention, liver transplantation, local ablation, and trans-arterial chemoembolization (TACE) radiation therapy are the main treatments for patients with HCC (Zhou et al., 2020). However, the prognosis of patients with HCC remains unfavorable. Drug resistance and precise therapeutic approaches are still challenging problems (Koulouris et al., 2021). Moreover, heterogeneity within a tumor is one of the main reasons contributing to currently ineffective therapies for most types of cancers, including HCC (Chan et al., 2022). Lesions within the same tumor may have different genomic alterations, biological behaviors, and local microenvironments, and may respond differently to a treatment. Even tumor cells in different areas of the same lesion may have different somatic mutations (Zhang et al., 2019). Due to tumor heterogeneity, there are significant differences in the prognosis of patients with HCC. Therefore, it is necessary to explore novel biomarkers that can accurately predict prognosis and guide clinical management for patients with HCC.

The ubiquitin-proteasome system (UPS) plays an essential role in maintaining cellular protein homeostasis through the degradation of short-life, misfolded, or non-essential cellular proteins (Colberg et al., 2020). UPS is a complex containing E1, E2, and E3 ubiquitinating enzymes and deubiquitinating enzymes as well as 26 S proteasome. It has been found that UPS is involved in various biological processes including in cell cycle, apoptosis, autophagy, epigenetic regulation, signaling transduction, and inflammatory and immune response (Zhou et al., 2019) (Çetin et al., 2021) (El Yaagoubi et al., 2021). Furthermore, the alteration of UPS leads to the initiation and progression of multiple diseases, such as kidney disease, Alzheimer’s disease, schizophrenia, and psoriasis (Yang et al., 2018) (Meyer-Schwesinger, 2019) (Al Mamun et al., 2020) (Luza et al., 2020). Recently, accumulating evidence has demonstrated that UPS dysregulation contributes to human malignancies progression (Yerlikaya et al., 2021). For instance, E3 ligases-mediated P53 degradation was clarified to be responsible for tumor development and progression (Bang et al., 2019). Targeting UPS may help to find potential therapeutic strategies for cancer patients (Zhang et al., 2020). Notably, a recent study has revealed the pivotal role of UPS in HBV viral replication and the pathogenesis of HCC (Kong et al., 2019). For example, ubiquitin-protein ligase E3 component N-recognin 7 (UBR7) is a key negative regulator of aerobic glycolysis and HCC oncogenesis (Zhao et al., 2022). However, the functional role and clinical value of UPS-related genes in HCC have not been fully investigated.

In this study, differentially expressed UPS genes (DEUPS) were screened based on the LIHC-TCGA dataset. Key UPS-associated genes were selected using univariate Cox regression and LASSO regression analyses. Next, we constructed a UPS-based prognostic risk model to accurately predict the clinical outcome of HCC. Furthermore, clinicopathologic characteristics, enrichment pathways, immune features, and drug sensitivity were further evaluated between the two risk groups. Collectively, our study sheds deeper insights into the underlying mechanisms of HCC pathogenesis and facilitates clinical decision-making based on targeting the UPS to manage HCC.

## Results

### Identification and functional enrichment analysis of DEUPS in HCC

**FIGURE 1:**
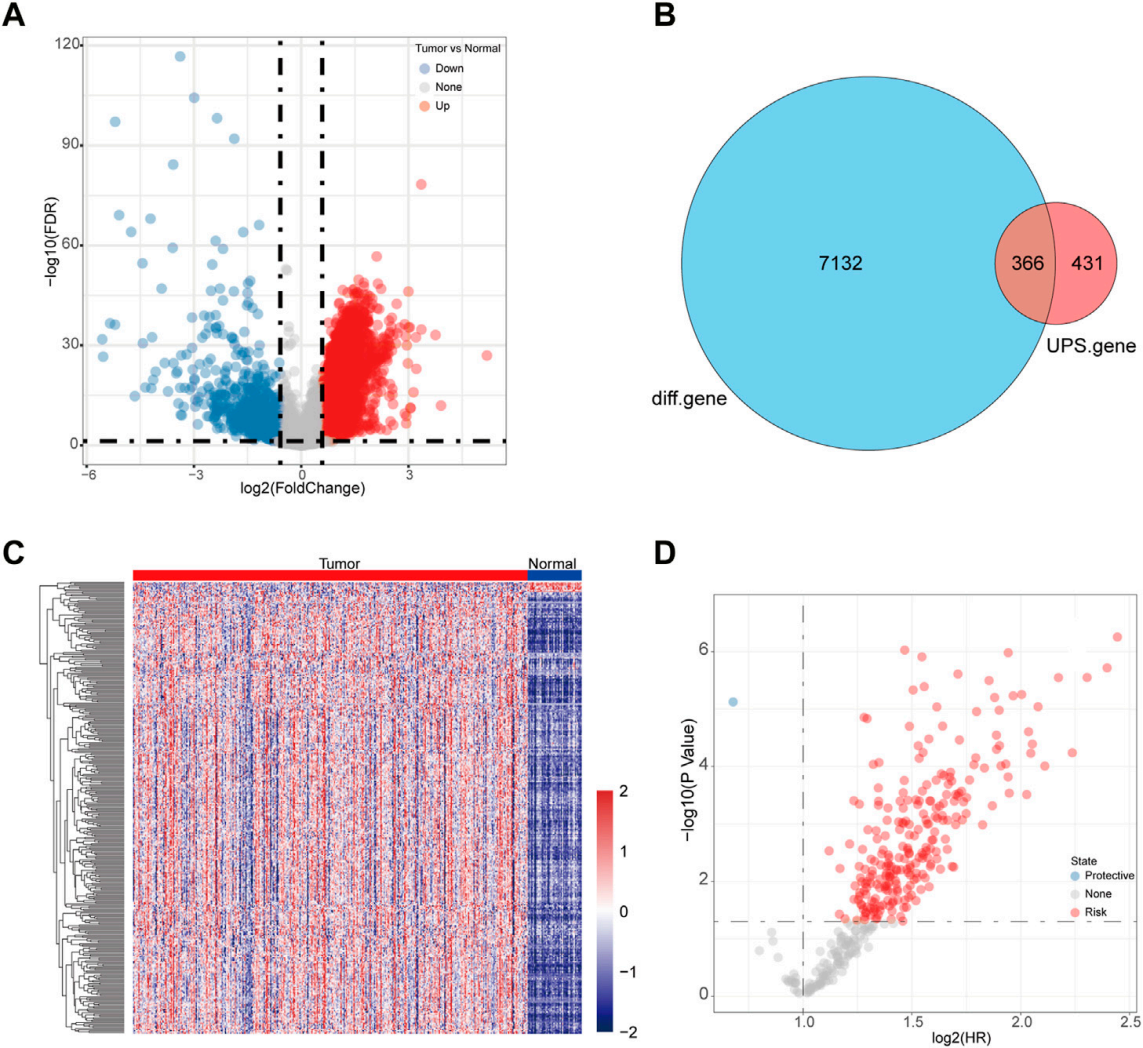
Identification of DEUPS in HCC. **(A)** Volcano plots displayed 6,682 upregulated and 816 downregulated genes between HCC samples and adjacent normal samples. **(B)** Venn diagram of overlap analyses for differential genes and UPS genes. **(C)** Heatmap showing the expression pattern of DEUPS between HCC samples and adjacent normal samples. **(D)** Scatter plot of univariate Cox regression analyses for screening prognosis-associated genes.

We first analyzed the differentially expressed genes based on the LIHC-TCGA datasets. A total of 6,682 upregulated genes and 816 downregulated genes were identified in HCC (Figure 1A). Next, 366 differentially expressed UPS (DEUPS) were filtered out through overlap analysis of the differentially expressed genes and UPS-related genes (Figure 1B). Then, the expression pattern of these DEUPS between HCC samples and adjacent normal samples was visualized, as shown in Figure 1C. Through screening prognosis-related genes by univariate Cox regression analysis, one protective gene and 249 risk genes were finally identified in HCC (Figure 1D).

**FIGURE 2:**
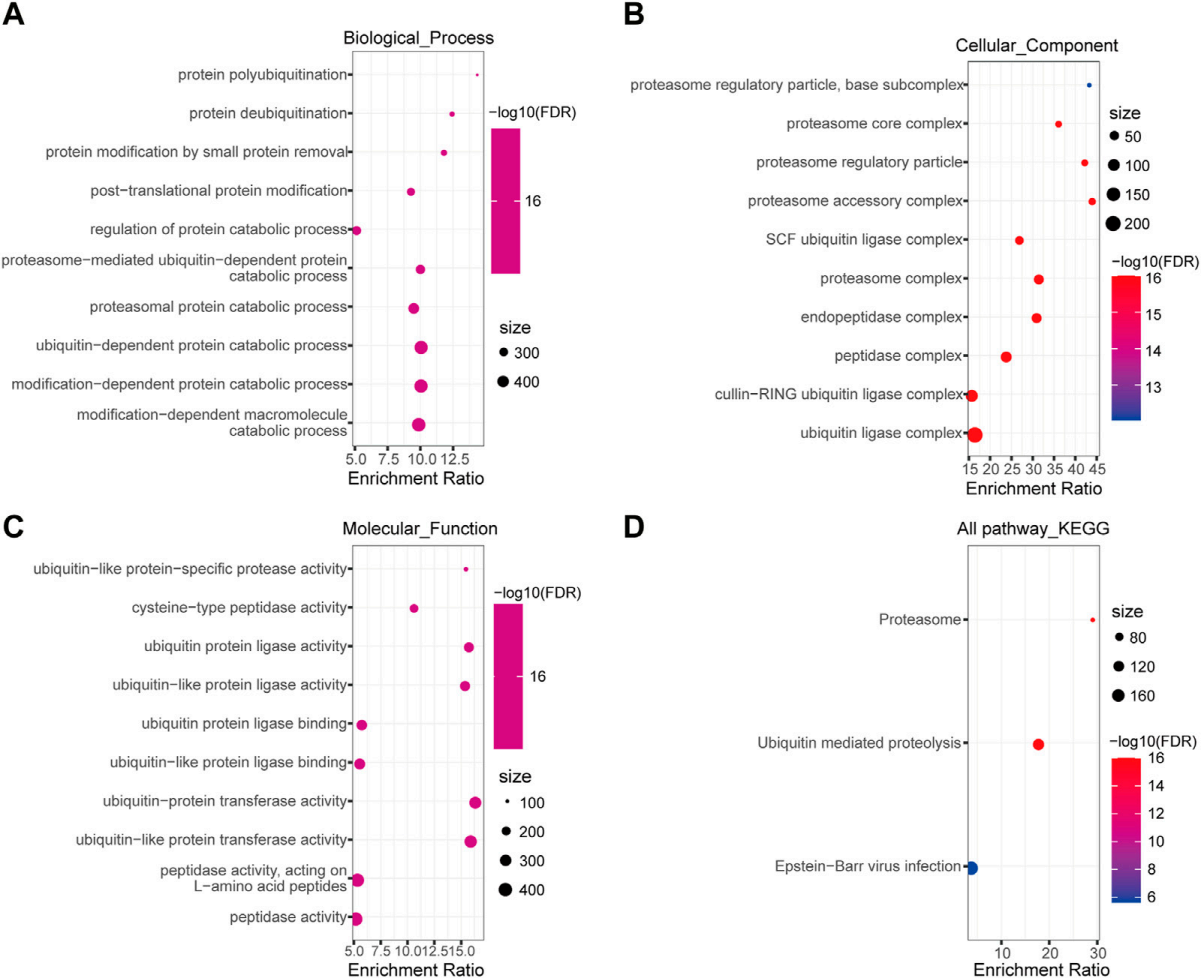
Enrichment analyses of DEUPS in HCC. **(A–C)** Top 10 terms in BP, CC, and MF categories. **(D)** KEGG Pathway analyses of DEUPS.

To gain insights into the biological function of DEUPS, GO and KEGG enrichment analyses were performed using 366 DEUPS. Under FDR <0.05, 119 items in biological process (BP), 26 items in cellular component (CC), 37 items in molecular function (MF), and 3 KEGG pathways were identified. The top 10 GO terms in BP, CC, and MF were shown (Figures 2A–C). KEGG analysis displayed that DEUPS were mainly enriched in the proteasome, ubiquitin-mediated proteolysis, and Epstein-Barr virus infection pathways in HCC (Figure 2D). This finding revealed a potential role of UPS in hepatocarcinogenesis.

### Development and verification of UPS-based prognostic model in HCC

**FIGURE 3:**
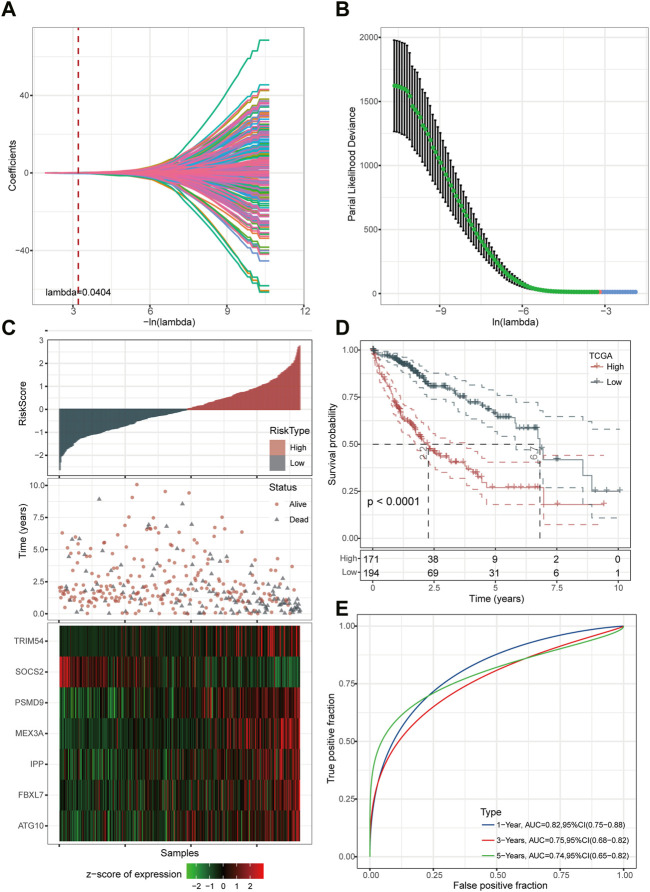
Determination of key genes for risk model construction. **(A)** Independent variable coefficients. **(B)** 10-fold cross-validation determining the confidence interval under each lambda.2 **(C)** Distribution of seven UPS-based signatures. **(D)** Kaplan-Meier curves of high- and low-risk patients in the TCGA cohort. **(E)** ROC curves with AUCs for 1-year, 3-year, and 5-year OS.

Firstly, we reduced the gene number for further analysis based on LASSO regression analyses. It was discovered that as lambda increased, the number of independent variable coefficients steadily tended toward zero (Figure 3A). Figure 3B presents the confidence interval for each lambda under the results of 10-fold cross-validation. Through further stepwise multivariate regression analyses with stepAIC, seven genes were selected for the risk model construction using the formula: RiskScore = 0.398*ATG10 + 0.193*FBXL7 + 0.282*IPP + 0.191*MEX3A-0.415*SOCS2 + 0.096*TRIM54 + 0.563*PSMD9.

**FIGURE 4:**
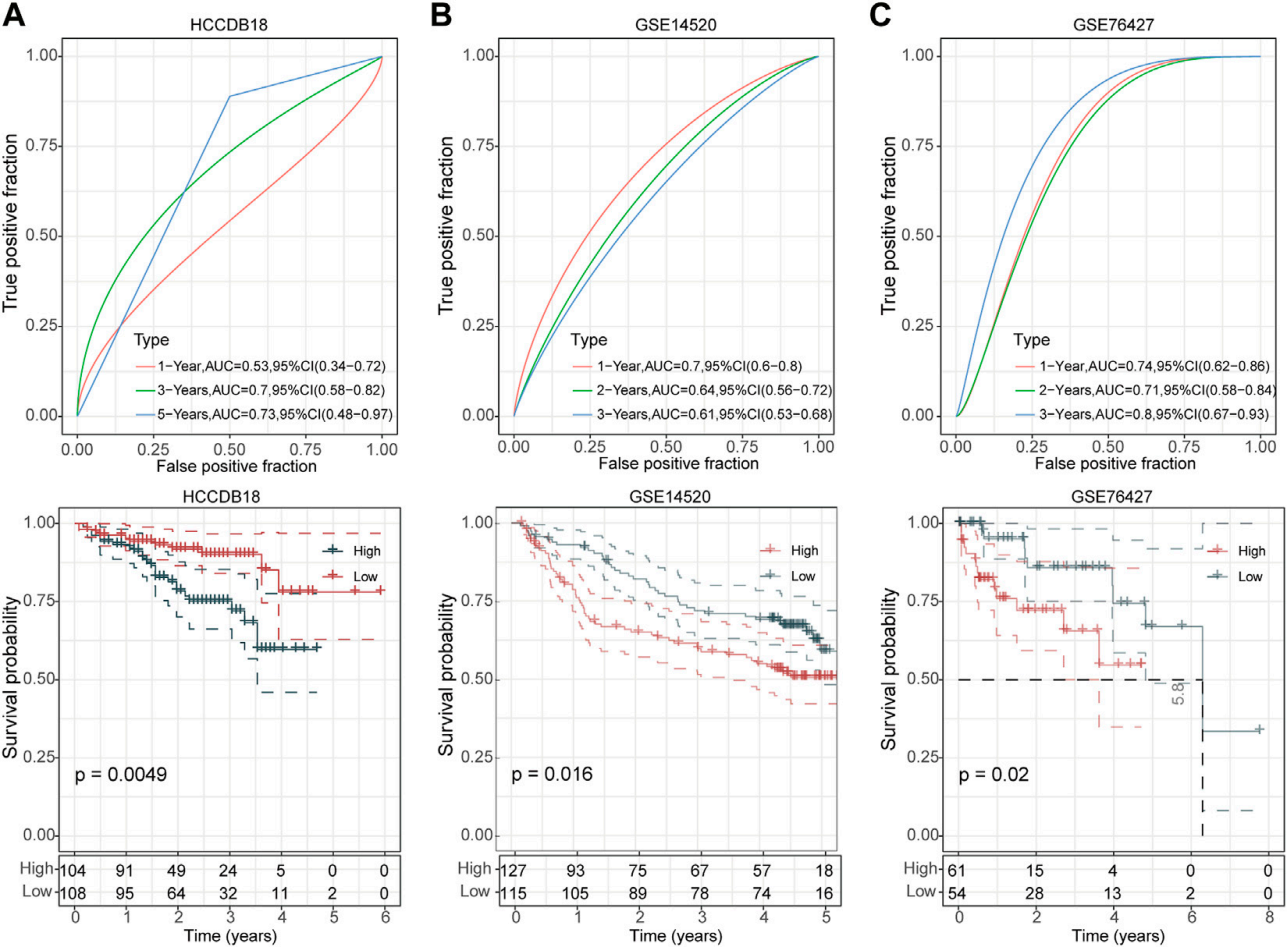
Validation of UPS-based prognostic model in three independent datasets. ROC analyses of the risk model for 1-year, 3-year, and 5-year OS (up) and Kaplan-Meier curves of high- and low-risk patients (down) in the HCCDB18 cohort **(A)**, GSE14520 cohort **(B)**, and GSE76427 cohort **(C)**, respectively.

We then divided the TCGA-LIHC cohort’s samples into high-risk and low-risk groups based on the risk scores (Figure 3C). Kaplan-Meier survival analysis showed that HCC patients with high-risk scores had a worse prognosis in comparison to those patients showing a low-risk score (*p* < 0.0001) (Figure 3D). Furthermore, the area under the receiver operating characteristic curve (ROC) was 0.82 (95% CI, 0.75–0.88) in predicting 1-year OS, 0.75 (95% CI, 0.68–0.82) in predicting 3-year OS, and 0.74 (95% CI, 0.65–0.82) in predicting 5-year OS. (Figure 3E). Then, we validated the robustness of this risk model in the HCCDB18, GSE14520, and GSE76427 cohorts and found a strong predictive performance of this risk model (Figures 4A–C). The KM survival analysis in 32 pan-cancer showed that the RiskScore could predict prognosis in 23 cancers (Supplementary Figure S1). Those results indicated the robustness of the RiskScore.

### Correlation analyses of the prognostic risk model with clinicopathologic characteristics

**FIGURE 5:**
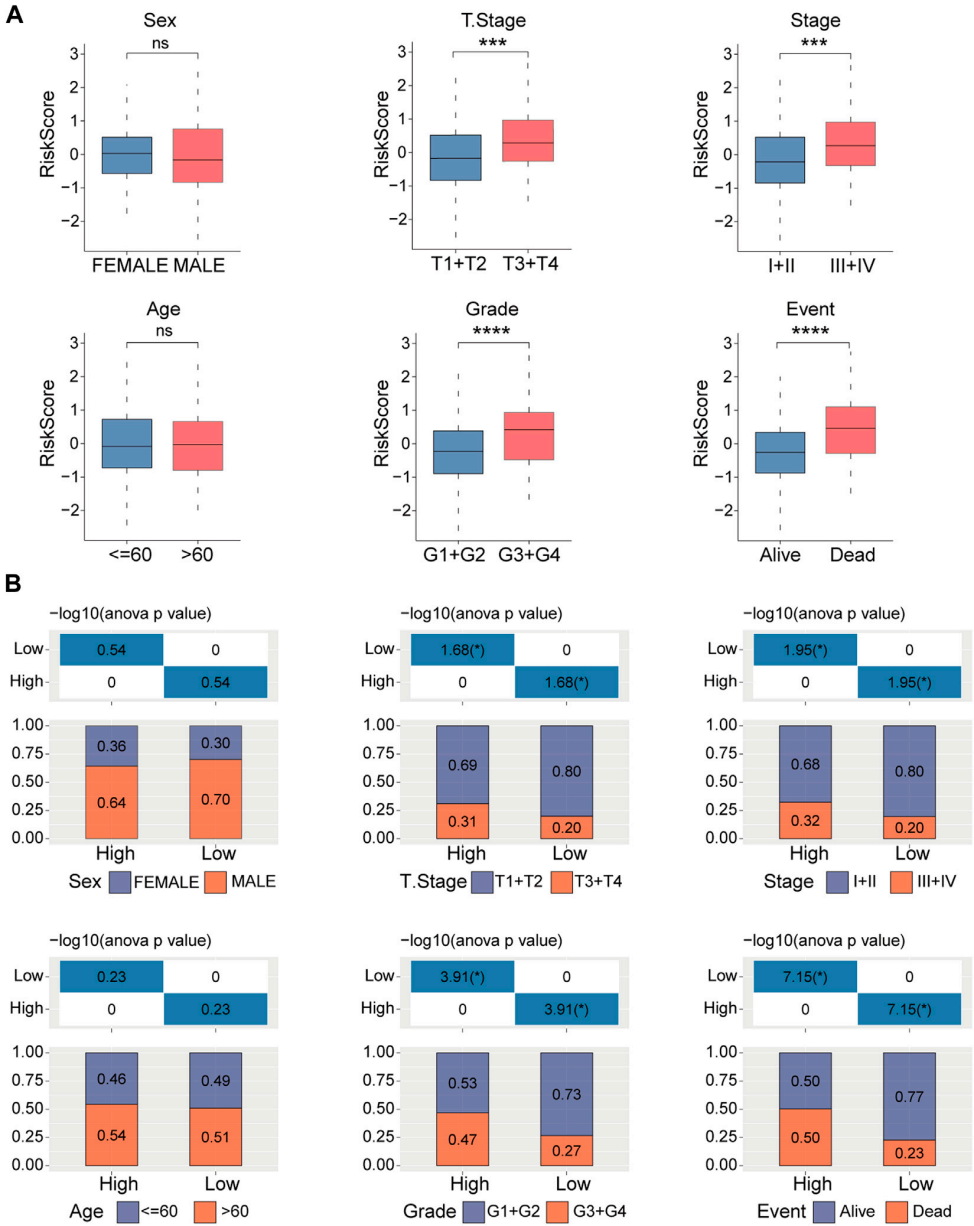
Clinicopathologic characteristics analyses in high- and low-risk groups. **(A)** Distribution of the risk score in different clinicopathologic characteristics including sex, age, tumor size (T), TNM stage, Grade, and survival status. **(B)** Distribution of the clinicopathologic characteristics in the high- and low-risk groups. ns, no significance, ^***^ *p* < 0.001, and ^****^ *p* < 0.0001.

Next, we examined the distribution of the risk score across various clinicopathological characteristics in the TCGA-LIHC cohort. We observed a significant increase in the risk score among patients with larger tumor sizes, advanced TNM stage, higher tumor grade, and poorer clinical outcomes (Figure 5A). Similar results are shown in Figure 5B. These findings suggest that patients with higher risk scores display more advanced clinicopathological features and have a poorer prognosis in HCC. The copy number of six genes manifested varying degrees of amplification or deletion. The FBXL7 gene had the highest mutation (Supplementary Figure S2).

### Pathway enrichment analysis of UPS-based prognostic risk signature

**FIGURE 6:**
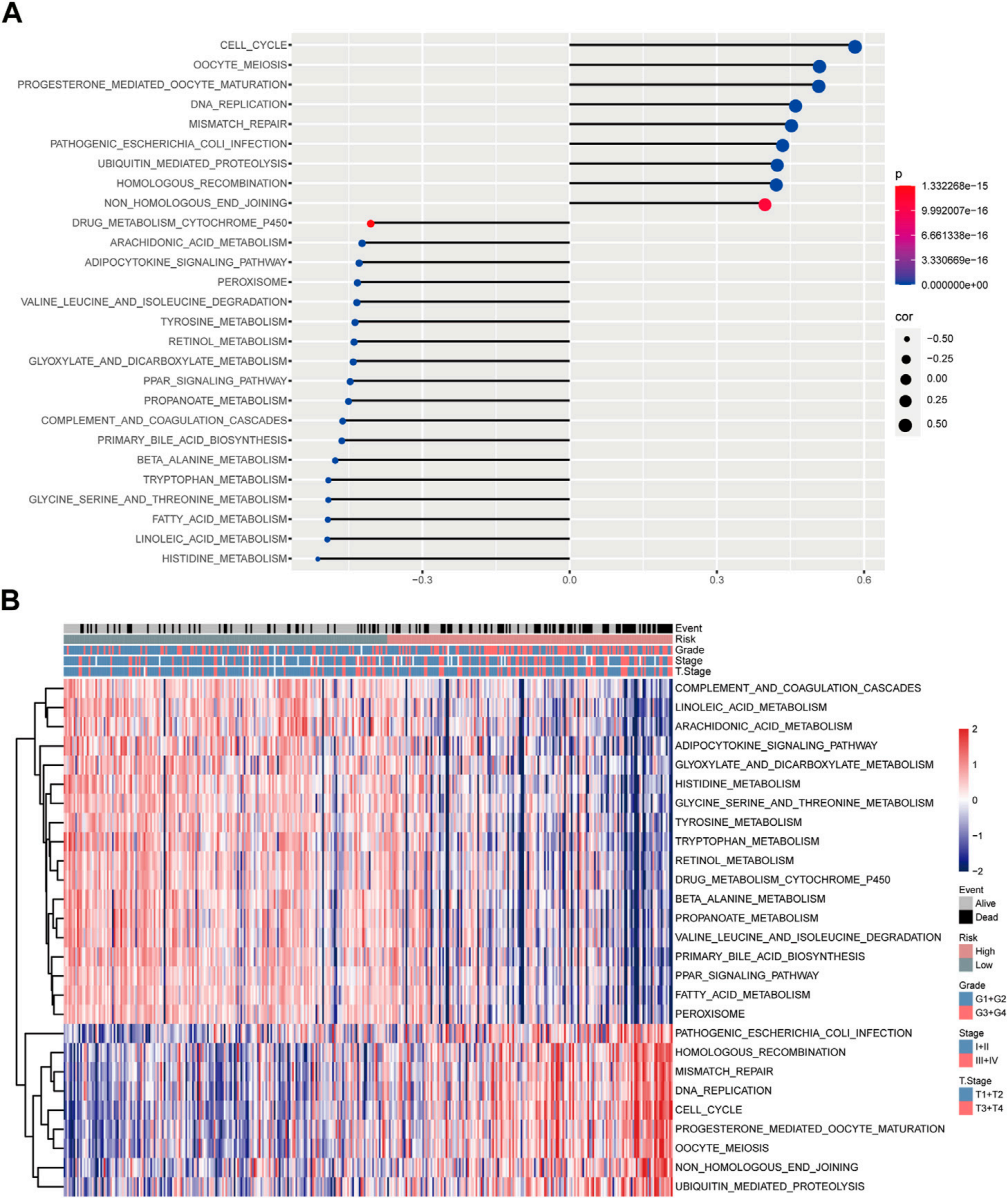
Potential regulatory pathways of the risk model. **(A)** Correlation analyses between risk score and enriched pathways. **(B)** Distribution of the enriched pathways in high- and low-risk groups are shown in the Heatmap.

Pathway analyses deciphered that several pathways, including DNA_REPLICATION, OOCYTE_MEIOSIS, CELL_CYCLE, UBIQUITIN_MEDIATED_PROTEOLYSIS, and HOMOLOGOUS_RECOMBINATION, and MISMATCH_REPAIR, were positively correlated with the risk score, while some metabolism-associated pathways, such as DRUG_METABOLISM_CYTOCHROME_P450, PRIMARY_BILE_ACID_METABOLISM, FATTY_ACID_METABOLISM, and ARACHIDONIC_ACID_METABOLISM, were negatively correlated with the risk score (Figure 6A). The heatmap showed pathways enriched in low- and high-risk groups (Figure 6B). These results suggested the underlying mechanism of UPS involved in HCC.

### Assessment of the prognostic risk model on immunophenotype

**FIGURE 7:**
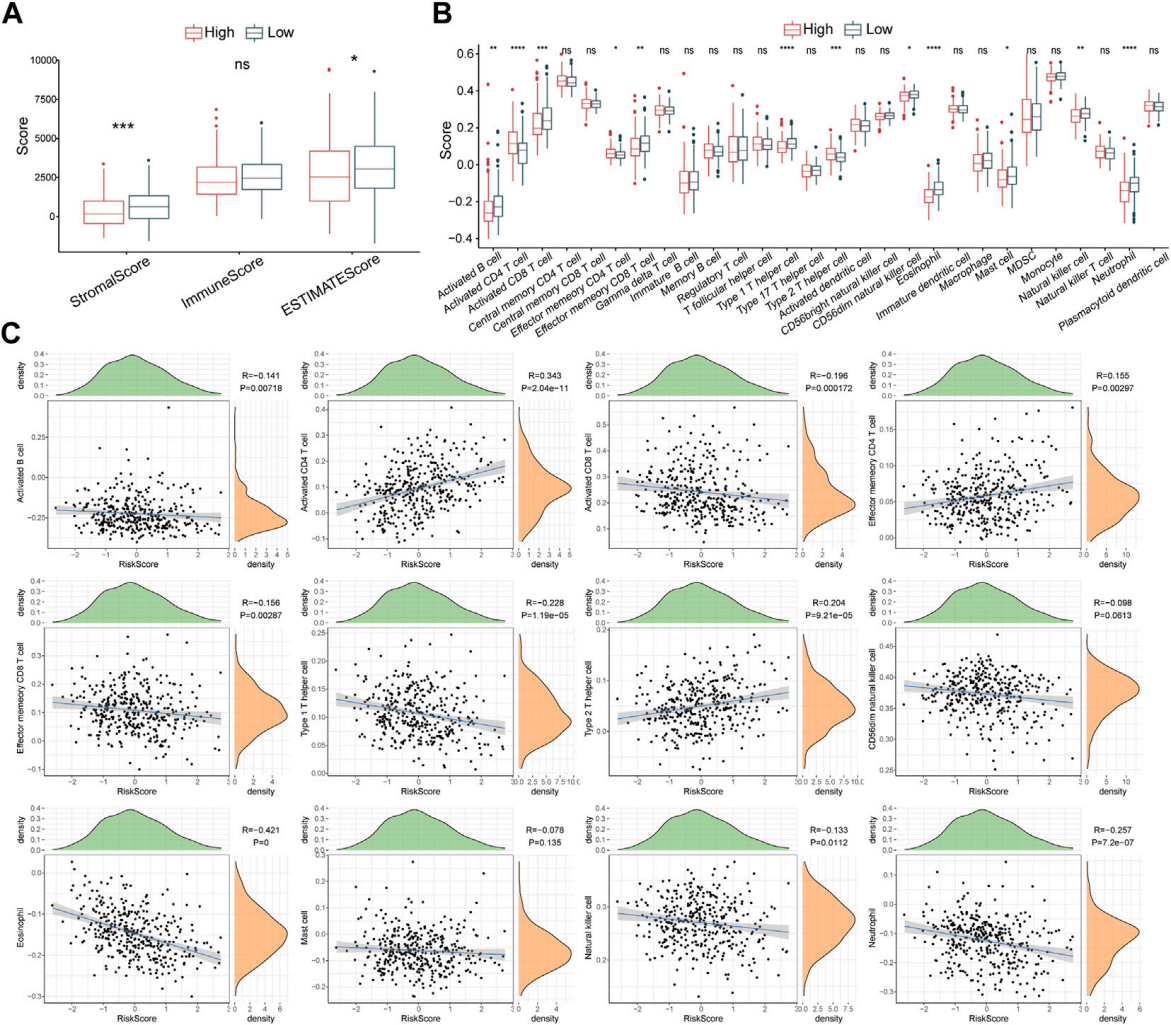
Immune characteristics of the risk model. **(A)** The Stromal score, Immune score, and ESTIMATES score in high- and low-risk groups. **(B)** The enrichment scores of 28 immune cells in high- and low-risk groups **(C)** Scatter plots display the correlation between risk score and 12 immune cells. ns, no significance, ^*^ *p* < 0.05, ^**^ *p* < 0.01, ^***^ *p* < 0.001, and ^****^ *p* < 0.0001.

The ESTIMATE algorithm was utilized to assess the immune characteristics of the risk model. The results revealed that patients with HCC from the high-risk group had lower stromal scores and ESTIMATE scores (Figure 7A). ssGSEA showed a significant differentiation in immunophenotype between the two risk groups. Notably, effector memory CD8^+^ T cell, activated B cell, natural killer cell, neutrophil, activated CD8^+^ T cell, CD56^dim^ natural killer cell, eosinophil, and type 1 T helper cell were significantly increased in the low-risk group, while the type 2 T helper cell, activated CD4^+^ T cell, and effector memory CD4^+^ T cell were elevated in high-risk patients (Figure 7B). Further correlation analyses showed that the risk score was negatively associated with effector memory CD8^+^ T cell, activated B cell, neutrophil, type 1 T helper cell, natural killer cell, eosinophil, CD8^+^ T cell, and CD56^dim^ natural killer cell, whereas positively associated with type 2 T helper cell, activated CD4^+^ T cell, and effector memory CD4^+^ T cell (Figure 7C). In addition, 15 immune-related pathways were downloaded from KEGG, and the scores were calculated by ssGSEA analysis. The score differences of 15 pathways between high- and low-risk groups indicated that three immune pathways had significant differences between high- and low-risk groups (Supplementary Figure S3).

### Evaluation of the prognostic risk model on drug sensitivity

**FIGURE 8:**
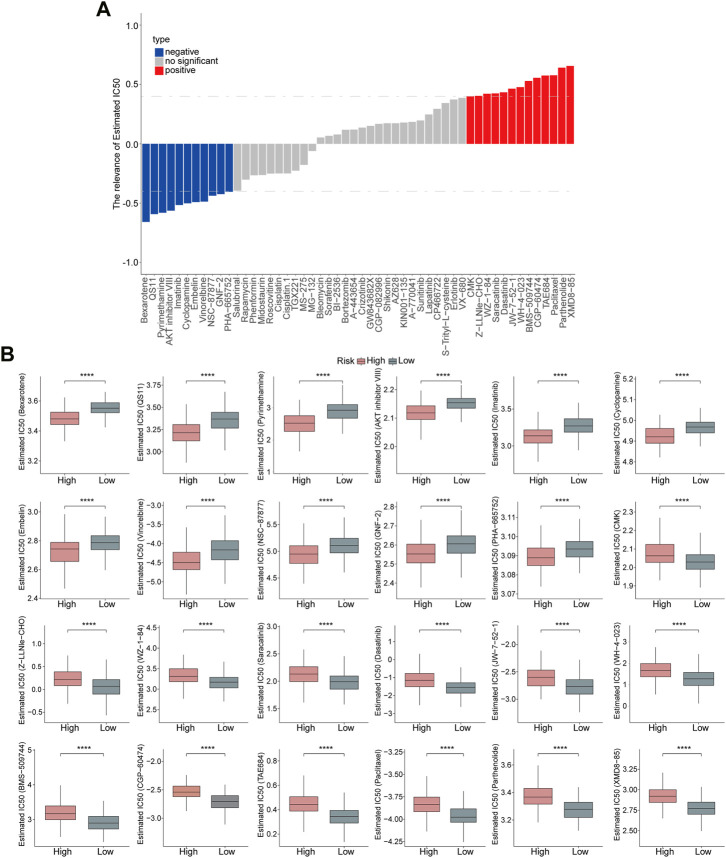
Evaluation of the risk model on drug sensitivity. **(A)** Correlation analyses between risk score and chemotherapeutic drugs are shown in the Histogram. **(B)** Estimated IC50 values of chemotherapeutic drugs between the high- and low-risk groups. *p* < 0.0001.

Subsequently, the sensitivity of high- and low-risk groups to chemotherapeutic drugs was evaluated. The risk score showed a positive correlation with XMD8-85, Parthenolide, Paclitaxel, TAE684, CGP-60474, BMS-509744, WH-4-023, JW-7-52-1, Dasatinib, Saracatinib, WZ-1-84, Z-LLNle-CHO, and CMK, but negative correlation with PHA-665752, GNF-2, NSC-87877, Vinorelbine, Embelin, Cyclopamine, Imatinib, AKT inhibitor VIII, Pyrimethamine, QS11, and Bexarotene. (Figure 8A). Moreover, high-risk patients were more sensitive to Bexarotene, QS11, Pyrimethamine, AKT inhibitor VIII, Imatinib, Cyclopamine, Embelin, Vinorelbine, NSC-87877, GNF-2, PHA-665752, while low-risk patients were more sensitive to CMK, Z-LLNle-CHO, WZ-1-84, Saracatinib, Dasatinib, JW-7-52-1, WH-4-023, BMS-509744, CGP-60474, TAE684, Paclitaxel, Parthenolide, and XMD8-85 (Figure 8B), which revealed that this risk model could significantly distinguish the clinical therapeutic response.

### Establishing a nomogram integrated with a prognostic risk score and clinicopathological features

**FIGURE 9:**
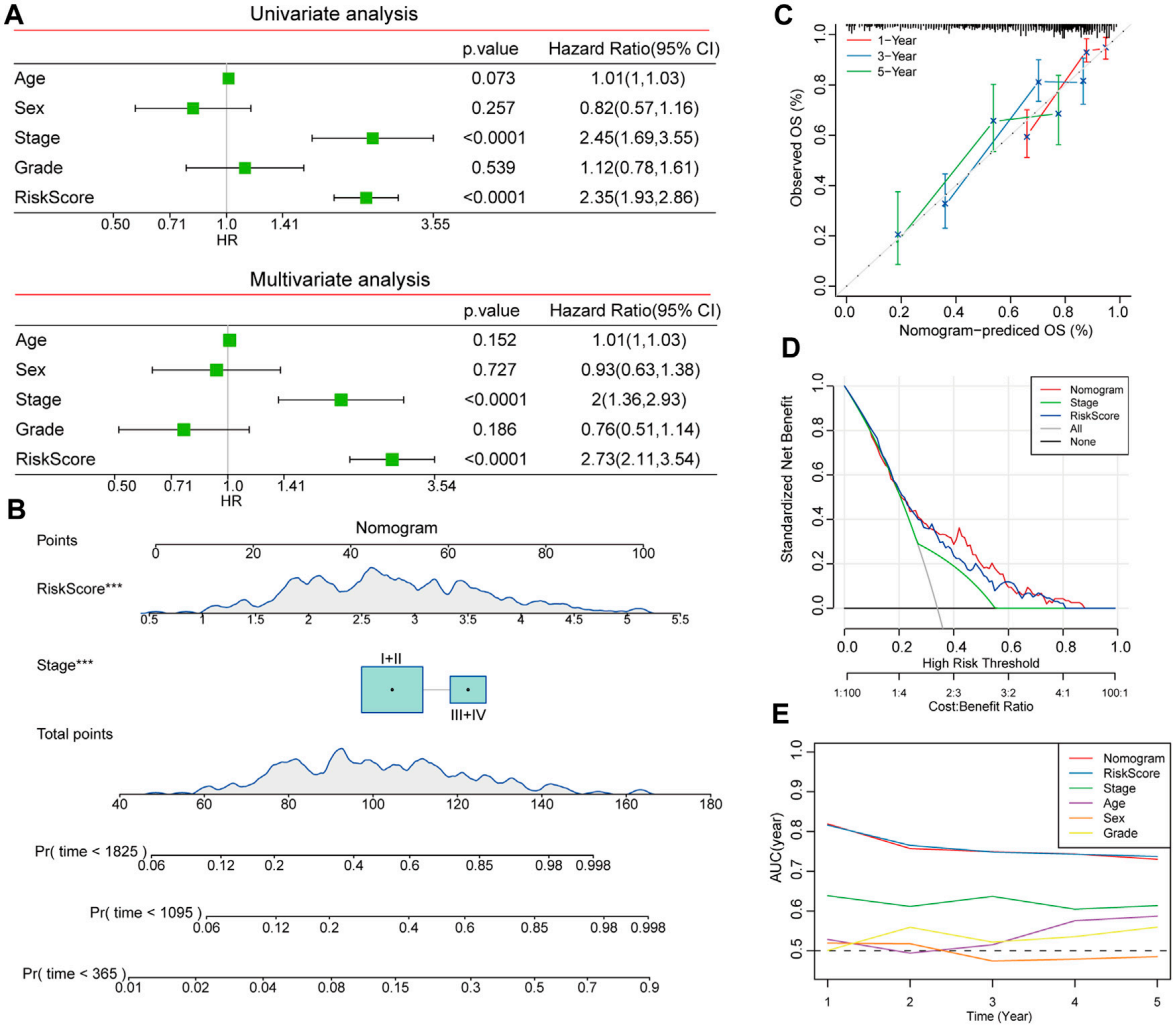
Construction of nomogram based on risk score and clinicopathological characteristics. **(A)** Independent prognostic factors were determined by univariate and multivariate Cox regression analyses. **(B)** Construction of nomogram to assess the predictive efficiency of risk score. **(C)** Calibration curves for nomogram predicted OS and observed OS for 1 year, 3 years, and 5 years. **(D)** DCA curves showed the reliability of the nomogram. **(E)** AUCs of different clinicopathologic characteristics for 1, 2, 3, 4, and 5-year OS.

Using univariate and multivariate Cox regression analyses, we identified risk scores combined with the TNM stage as independent prognostic markers of patients with HCC (Figure 9A). We evaluated the predictive effectiveness of this risk model using a nomogram, and the risk score showed the highest influence on survival prediction (Figure 9B). The anticipated calibration curves for 1-year, 3-year, and 5-year were nearly identical to the reference curves, indicating a strong predictive ability of the nomogram analyses (Figure 9C). Furthermore, the DCA results demonstrated that both the nomogram and risk score had the strongest survival prediction ability (Figures 9D, E).

## Discussion

The UPS serves a critical role in cancer initiation and progression. Emerging evidence has highlighted the role of UPS as a promising therapeutic target for cancer management. Recently, UPS-based prognostic signatures have been identified in pancreatic cancer, head and neck squamous cell carcinoma (HNSCC), and papillary renal cell carcinoma (Lankes et al., 2020; Wang et al., 2021; Zhang et al., 2022). The present study screened DEUPS and developed a UPS-based prognostic risk model that could predict the prognosis and anti-tumor drug sensitivity of patients with HCC.

In the present study, we established a UPS-based prognostic signature in HCC including ATG10, FBXL7, IPP, MEX3A, SOCS2, TRIM54, and PSMD9. The risk model exhibited a strong performance in survival prediction. Previous studies have identified that ATG10 was associated with autophagy, immune response, and tumor metastasis in HCC (Zhang J. et al., 2021; Chen et al., 2021; Xu et al., 2021), which suggested that UPS-related genes might contribute to HCC development through regulating biological process. FBXL7, an F-box protein that binds to substrates via SKP1-Cullin-1-F-box (SCF) E3 ubiquitin ligase, could enhance polyubiquitylation and degradation of substrates in tumor occurrence and aggression (Wang et al., 2022). Additionally, FBXL7 reduces chemotherapy resistance by promoting the ubiquitination and degradation of survivin (Dong et al., 2022). IDO-targeting PROTAC peptide (IPP), generated and activated from UPS, leads to the activation of effector T cells that could further suppress tumor growth and metastasis (Zhang C. et al., 2021). MEX3A, an RNA-binding ubiquitin ligase, has been verified to be involved in glioblastoma multiforme initiation and progression (Bufalieri et al., 2020). Furthermore, SOCS2 enhances the radiotherapy sensitivity of patients with HCC through mediated SLC7A11 ubiquitination (Chen et al., 2022). Furthermore, TRIM54 was identified as an oncogene in HCC (Zhu et al., 2021). Köster et al. have demonstrated that a high level of PSMD9 is strongly associated with clinical relapse after radiotherapy in patients with cervical cancer (Köster et al., 2020). Collectively, UPS-related molecules might contribute to hepatocarcinogenesis.

High immune cell infiltration and immunosuppression characterize the tumor microenvironment (TME), which represents a critical prognostic factor (Cariani and Missale, 2019). Natural killer cells are considered the first-line effector in innate immunity that can shape the TME of HCC and exert cytotoxic effects (Chen et al., 2023). Immunotherapeutic strategies targeting natural killer cells were evidenced as promising approaches to improve the clinical outcomes of patients with HCC (Habif et al., 2019). To induce an adaptive immune response in HCC, activated dendritic cells present process antigens that bind to class II Human Leukocyte Antigen molecules to naïve CD4^+^T cells, and then provoke the differentiation of CD4^+^ T cells into type 1 T helper cells, leading to differentiation into effector CD8^+^ cytotoxic T lymphocytes (Cariani and Missale, 2019). It has been reported that the abundance of intertumoral CD3^+^ and CD8^+^ cytotoxic T cells was strongly associated with the relapse-free survival rate in HCC (Gabrielson et al., 2016). Moreover, tumor-specific CD8^+^ T cells are crucial in immunotherapies (Hofmann et al., 2021). In this study, we observed that tumor-inhibiting associated immune cells were significantly increased in low-risk patients, suggesting that UPS-related risk genes might facilitate reshaping the evolution of HCC through regulating immune cell infiltration.

Increasing evidence has clarified the role of UPS in tumor cell proliferation (Wei et al., 2022). In this study, we found that UPS-based risk score was significantly positively correlated with DNA_REPLICATION, OOCYTE_MEIOSIS, CELL_CYCLE, MISMATCH_REPAIR, UBIQUITIN_MEDIATED_PROTEOLYSIS, and HOMOLOGOUS_RECOMBINATION. The results indicated that these seven UPS-based signatures were involved in HCC progression via regulating cell cycle and DNA repair pathways.

In the present study, we also performed drug sensitivity analyses and the results showed that these drugs were closely associated with the risk score, which indicated the potential clinical value of UPS in HCC treatment. Moreover, patients with high-risk scores were more sensitive to anti-tumor drugs such as Bexarotene, QS11, Pyrimethamine, AKT inhibitor VIII, Imatinib, Cyclopamine, Embelin, Vinorelbine, NSC-87877, GNF-2, and PHA-665752, while patients with low-risk scores were more sensitive to CMK, Z-LLNle-CHO, WZ-1-84, Saracatinib, Dasatinib, JW-7-52-1, WH-4-023, BMS-509744, CGP-60474, TAE684, Paclitaxel, Parthenolide, and XMD8-85. Our findings might offer new ideas for HCC management and guide the clinical personalized therapeutic strategies.

However, there are several limitations to this study. Firstly, the expression profiles of patients with HCC were retrieved from public databases including TCGA, HCCDB, and GEO. Therefore, the reliability of this signature needs to be validated by further prospective studies with a larger sample size. Additionally, functional experiments should be conducted to investigate the detailed mechanism of seven UPS-related genes. Further, the reliability of this prognostic signature should be iteratively improved with long-term clinical use.

## Conclusion

In summary, we identified seven UPS-based signatures that can accurately predict the clinical outcome and drug sensitivity of patients with HCC. Meanwhile, the UPS-based signatures might reveal the underlying mechanism of hepatocarcinogenesis and provide basic evidence for personalized therapies for patients with HCC.

## Material and methods

### Datasets

Gene expression profiles and survival data of patients with HCC were downloaded from The Cancer Genome Atlas (TCGA) database (Tumor, *n* = 365, Normal, *n* = 50) and Hepatocellular Carcinoma Database (HCCDB) (Tumor, *n* = 212) (Lian et al., 2018). Gene expression profiles of 242 HCC samples in GSE14520 and 115 HCC samples in GSE14520 were downloaded from the Gene-Expression Omnibus (GEO) database. Additionally, a total of 804 UPS-related gene datasets were collected according to a previous study (Wang et al., 2021).

### Differentially expressed genes analyses

To screen the differentially expressed UPS (DEUPS) between HCC samples and adjacent normal samples, the “limma” package (Ritchie et al., 2015) in R software was used. Genes with fold change (FC) > 1.5 or <0.67, and false discovery rate (FDR) < 0.05 were considered differentially expressed. Furthermore, univariate Cox regression analyses were employed using the “survival” package (Therneau and Lumley, 2015) to identify prognosis-associated genes.

### Functional enrichment analyses

Gene Ontology (GO) and KEGG enrichment analyses were conducted using the “WebGestaltR” package (Liao et al., 2019). The GO functional enrichment included cellular component (CC), molecular function (MF), and biological process (BP) categories. Candidates with FDR <0.05 were considered as having significantly enriched pathways.

### Construction and validation of UPS-based prognostic model

To identify key UPS-related genes for prognostic model construction, the LASSO regression analyses were performed using the “glmnet” package (Hastie et al., 2021). Subsequently, we performed stepwise multivariate regression analyses with stepwise Akaike information criterion (stepAIC) to determine key prognostic genes.

The risk score of individuals in the TCGA cohort was calculated as the following formula: RiskScore = 0.398*ATG10 + 0.193*FBXL7+0.282*IPP + 0.191*MEX3A-0.415*SOCS2 + 0.096*TRIM54 + 0.563*PSMD9. “timeROC” package was used for receiver operating characteristic (ROC) analyses (Blanche, 2015). Areas under the ROC curve (AUCs) for 1-year, 3-year, and 5-year OS were evaluated. After standardization, patients in the TCGA cohort were grouped into high-risk (z score >0) and low-risk (z score <0). To validate the robustness of the risk model, the seven UPS-based signature was evaluated in the HCCDB18, GSE14520, and GSE76427 cohorts.

### Pathway enrichment analyses

To evaluate the underlying regulatory pathways of the risk model, we downloaded KEGG-related datasets from the Gene Set Enrichment Analyses (GSEA) website and scored them using the “GSVA” package (Hänzelmann et al., 2013). Correlation analyses of risk scores and pathway scores were performed using the “Hmisc” package (Harrell Jr and Harrell Jr, 2019). Significant differential pathways were selected based on |cor| > 0.4 and *p* < 0.05.

### Immune characteristics analyses

Furthermore, the immune score was calculated in the TCGA cohort using the ESTIMATE algorithm, and the scores of 28 immune cells from published research (Charoentong et al., 2017) were calculated using the ssGSEA method. The relationship between risk score and 12 immune cells was determined by Spearman correlation analyses.

### Drug sensitivity evaluation of risk groups

The half-maximal inhibitory concentration (IC50) was analyzed using the “pRRophetic” package to evaluate the anti-neoplastic drug sensitivity of patients with HCC in different risk groups (Geeleher et al., 2014). Correlation analyses between the risk score and estimated IC50 were conducted and candidates with |cor| > 0.4 were selected as having significantly correlated pathways.

### Construction of the nomogram

Univariate and multivariate Cox regression analyses were performed to evaluate the predictive efficiency of the risk model. Thereafter, a calibration curve was generated to quantify the prediction accuracy of the nomogram. Decision curve analyses (DCA) were further utilized to assess the reliability of the nomogram.

### Statistical analyses

All the statistical analyses were completed by the R program (Version 4.0.2). The Wilcoxon rank-sum test and the Student’s *t*-test were used to compare the two groups. The Kaplan-Meier method and the log-rank test were used to compare the survival probability. *p* < 0.05 was considered as a statistical significance.

## Data Availability

The datasets presented in this study can be found in online repositories. The names of the repository/repositories and accession number(s) can be found in the article/Supplementary Material.
