# Supplementary figures and images for: Ubiquitin-proteasome system-based signature to predict the prognosis and drug sensitivity of hepatocellular carcinoma
Source: Front Pharmacol. 2023 Apr 25;14:1172908. doi: 10.3389/fphar.2023.1172908 (PMC10166894; doi:10.3389/fphar.2023.1172908)

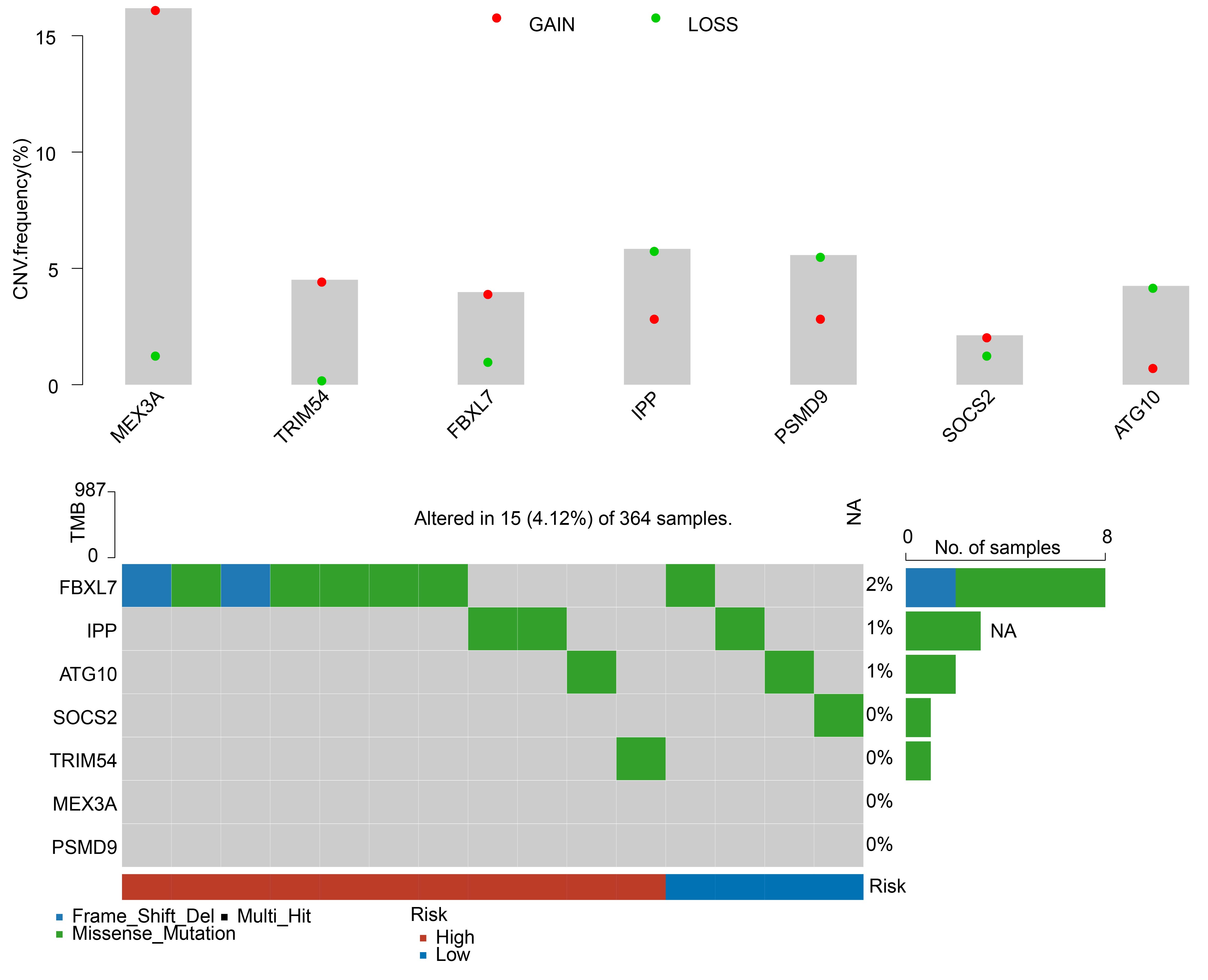

Supplement: Supplementary file 2 [file Image1.JPEG]

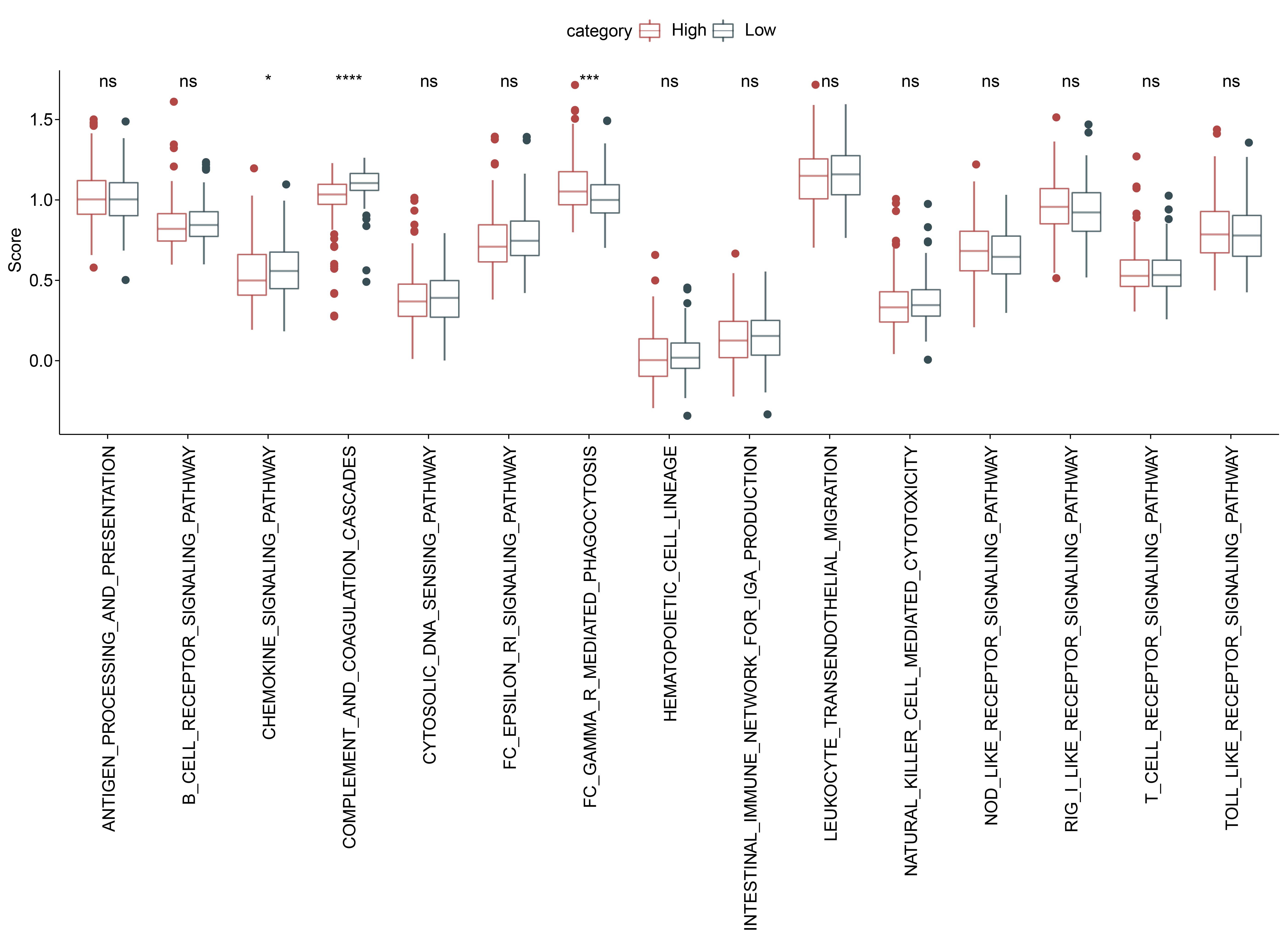

Supplement: Supplementary file 3 [file Image2.JPEG]
